# Predicting nosocomial lower respiratory tract infections by a risk index based system

**DOI:** 10.1038/s41598-017-15765-z

**Published:** 2017-11-21

**Authors:** Yong Chen, Xue Shan, Jingya Zhao, Xuelin Han, Shuguang Tian, Fangyan Chen, Xueting Su, Yansong Sun, Liuyu Huang, Hajo Grundmann, Hongyuan Wang, Li Han

**Affiliations:** 1 Chinese PLA Institute for Disease Control and Prevention, Beijing, China; 2 School of Public Health, Peking University, Beijing, China; 3 Department of Infection Prevention and Hospital Hygiene, Faculty of Medicine, University of Freiburg, Freiburg, Germany; 4 Department of Medical Microbiology, University Medical Center Groningen, Rijksuniversteit Groningen, Groningen, The Netherlands

## Abstract

Although belonging to one of the most common type of nosocomial infection, there was currently no simple prediction model for lower respiratory tract infections (LRTIs). This study aims to develop a risk index based system for predicting nosocomial LRTIs based on data from a large point-prevalence survey. Among the 49328 patients included, the prevalence of nosocomial LRTIs was 1.70% (95% confidence interval [CI], 1.64% to 1.76%). The areas under the receiver operating characteristic (ROC) curve for logistic regression and fisher discriminant analysis were 0.907 (95% CI, 0.897 to 0.917) and 0.902 (95% CI, 0.892 to 0.912), respectively. The constructed risk index based system also displayed excellent discrimination (area under the ROC curve: 0.905 [95% CI, 0.895 to 0.915]) to identify LRTI in internal validation. Six risk levels were generated according to the risk score distribution of study population, ranging from 0 to 5, the corresponding prevalence of nosocomial LRTIs were 0.00%, 0.39%, 3.86%, 12.38%, 28.79% and 44.83%, respectively. The sensitivity and specificity of prediction were 0.87 and 0.79, respectively, when the best cut-off point of risk score was set to 14. Our study suggested that this newly constructed risk index based system might be applied to boost more rational infection control programs in clinical settings.

## Introduction

Healthcare associated infection (HAI) represents a major public health problem from all around the world^1–3^. Patients with HAI might have prolonged hospital stays, and have high morbidity and mortality, thus adding economic burden on the healthcare system^4^. Pneumonia and other lower respiratory tract infections (LRTIs) were the most common type of HAIs^1,5^. According to a large multicenter epidemiological survey from China, 8,739 (59.55%) of 14,674 HAIs cases belonged to LRTI^6^.

It has been suggested that risk prediction model may have applications in identifying high-risk patients and evaluating the effectiveness of infection control measures^7–9^. Currently, some studies have identified the risk factors of nosocomial LRTIs, which include tracheal intubation, underlying chronic lung disease, supine body position, mechanical ventilation, thoracic or upper abdominal surgery, prior episode of a large volume aspiration, and age older than 70 years^10–12^. However, these findings are difficult to be applied for risk prediction. Risk index based method is therefore needed in terms of its simplicity and feasibility in predicting the probability of acquiring nosocomial LRTIs.

There are currently many risk index based systems in clinical field, such as acute physiology and chronic health evaluation II (APACHE II), therapeutic intervention scoring system (TISS) and simplified acute physiology score (SAPS)^13,14^, however, these systems are not targeted for HAIs. Up to date, there are some studies trying to evaluate the application of risk index in predication of HAIs^15^ or surgical site infections (SSI)^16^, but no study focused on the predication of nosocomial LRTIs.

The purpose of this study was to develop a risk index based system for predicting nosocomial LRTIs using data from a large point-prevalence survey of HAIs, and to evaluate its sensitivity and specificity in identifying infection.

## Methods

### Data source and study population

The data used in this study comes from a large one-day point-prevalence survey of HAIs between October 2014 and March 2015^17^. All variables for each patient were extracted from the individual report form of this survey, including demographic data, days of hospitalization on survey date, invasive procedures, use of antibiotics and underlying diseases. Underlying diseases for each patient were transformed into ICD-10-CM three-character categories code and treated as a binary variable. The detailed information for these variables was shown in Supplemental Table S1. 49328 cases of patients from 46 hospitals with completed information for all variables were included in this study. This study was approved by institutional review boards (IRB) of Academy of Military Medical Science. All methods were performed in accordance with the relevant guidelines and regulations. As all the data were analyzed anonymously, the IRB of Academy of Military Medical Science waived the informed consent requirement.

### Case definition

The definition of nosocomial LRTIs was in accordance with diagnosis guideline issued by the National Health and Family Planning Commission of the People’s Republic of China (NHFPC; formerly the Chinese Ministry of Health) in 2001. Nosocomial LRTIs, which comprise of the CDC categories of ‘pneumonia’ and ‘lower respiratory tract infection other than pneumonia’, refer to infection acquired after 48 hours’ admission, unless there is a clear incubation period for the infection. Ventilator-associated pneumonia (VAP) was excluded for analysis in this study.

### Logistic regression and fisher discriminant analysis

There’re several options for diagnostic and prognostic tasks in clinical medicine, of which we chose multivariate logistic regression and fisher discriminant analysis, both known as so-called white box models that allow an interpretation of model parameter^18^. For multivariate logistic regression, a backward selection algorithm was used, the coefficient (β) of the variables was estimated. The wald χ^2^ test was used to assess the covariate-adjusted *p* value. The *p* value for statistical testing of variable significance for exclusion from model is set to 0.05. Candidate risk factor variables are listed in Supplemental Table S1. For fisher discriminant analysis, those variables statistically significant in logistic regression analysis were used as input factors, and whether or not an individual has nosocomial LRTI is the classification factor. The area under the receiver operating characteristic (ROC) curve was calculated for both predictive models.

### Construction of risk index based system

The risk index based system was constructed by simplifying the parameters based on the logistic regression model with better ROC curve performance. A 10-fold cross validation scheme was applied to test the robustness of the logistic model. This means that we randomly divided the training data set into 10 partitions, applied the classification method 10 times to the data from 9 partitions, and used the respective 10^th^ partition to test the performance. After this series of 10 classification tasks, we derived a ROC curve figure using the mean of each performance parameter.

If the difference between ROC curves of original logistic model and 10-fold cross validation is small, we can deem that logistic is a robust model. Then the risk index was derived based on the original logistic model. We firstly determined the smallest absolute value *u* of the coefficients, then divided all the parameters by *u*, and rounded the results to the nearest integer. To further simplify risk index system, approximate index values were adjusted to the same score. One patient’s total risk score is calculated through adding the scores of all risk items of the patient.

The prevalence of LRTIs was calculated for patients with different risk scores. Different risk levels were determined according to the total risk scores of each patient. The sensitivity and specificity of different risk scores were calculated for determining the best cut-off point. The performance of cut-off point in predicting LRTIs was also evaluated for patients with different underlying diseases. All the data were analyzed with SAS 9.4. All tests were considered as significant at p < 0.05.

## Results

### The prevalence of LRTIs

Among the 49328 patients included in this study, there were 839 cases of nosocomial LRTIs, the overall prevalence was 1.70% (95% confidence interval [CI], 1.64% to 1.76%). The prevalence of nosocomial LRTIs among patients with different underlying diseases ranged from 0.2% to 50.0% (Supplemental Table S2). High prevalence was detected in patients with the following diseases: other respiratory disorders (17.3%), hydrocephalus (10.9%), nontraumatic intracerebral hemorrhage (10.3%), acute respiratory distress syndrome (7.1%), sequelae of cerebrovascular disease (5.9%), emphysema (5.8%), myeloid leukemia (5.5%) and lymphoid leukemia (5.3%). These diseases, which might jeopardize respiratory function, compromise the immune system or lead to patients’ prolonged stays in hospital, are considered to be significantly associated with the occurrence of LRTIs.

### Prediction of LRTIs by logistic regression and fisher discriminant analysis

**Figure 1:**
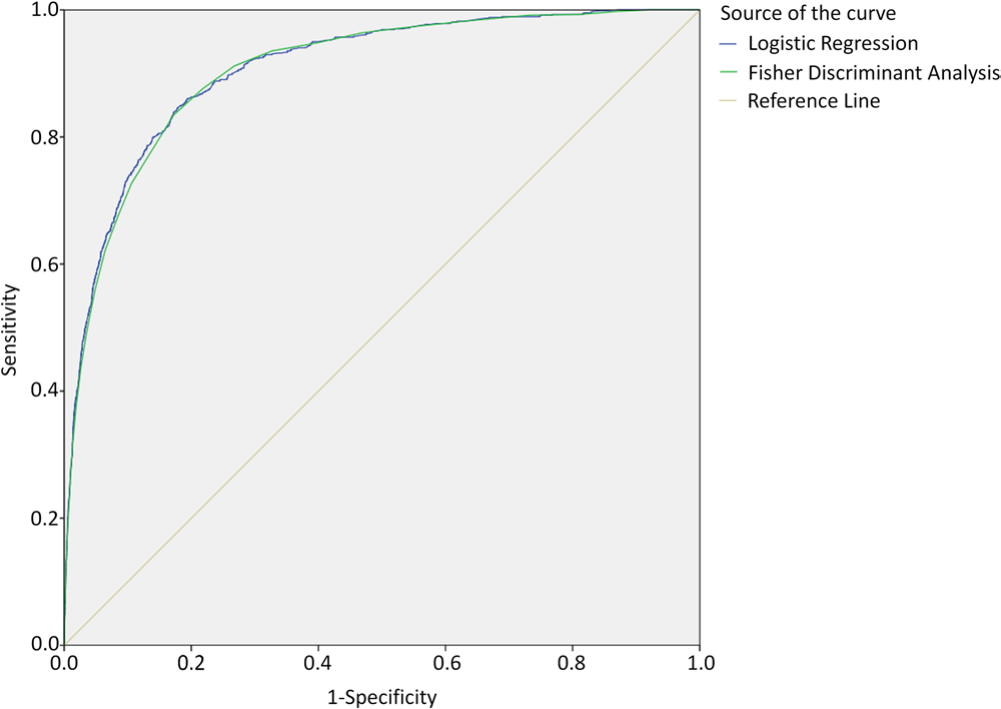
The ROC curves for predicting nosocomial lower respiratory tract infection derived from logistic regression and fisher discriminant analysis.

Logistic regression analysis showed that age, male, length of hospital stay, tracheotomy, artery or venous catheterization, urinary tract intubation are risk factors for nosocomial LRTIs. Prophylactic use of antibiotics can effectively reduce the risk of acquiring LRTIs. Judging from the estimated standardized regression coefficient, age has the strongest influence on whether a patient would acquire LRTI, for which the standardized coefficient is 0.36, followed by the length of hospital stay (0.31) and urinary tract intubation (0.22). Areas under the ROC curve for logistic regression and fisher discriminant analysis were 0.907 (95% CI, 0.897 to 0.917) and 0.902 (95% CI, 0.892 to 0.912), respectively. As shown in Fig. 1, there is little difference between the two statistical methods, especially for the curve on the upper left corner, where the best cut-off point most likely exists, suggesting that the predictive efficiency of the two models are almost the same.

### Prediction of LRTIs by risk index based system

**Figure 2:**
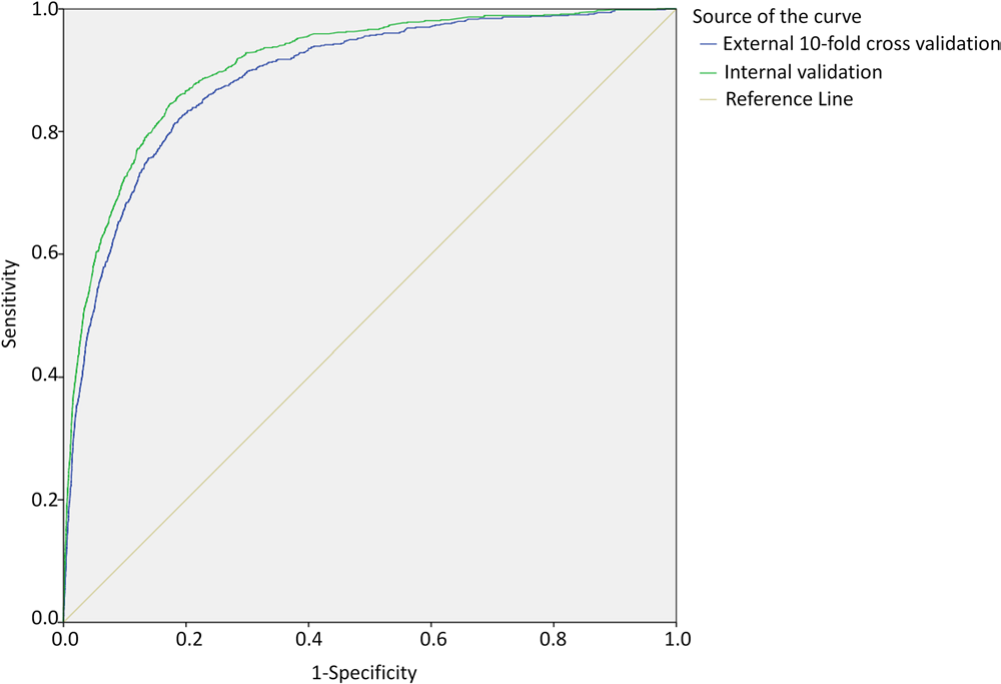
The ROC curves for predicting nosocomial lower respiratory tract infections derived from internal validation and external 10-fold cross validation scheme based on logistic regression model.

**Figure 3:**
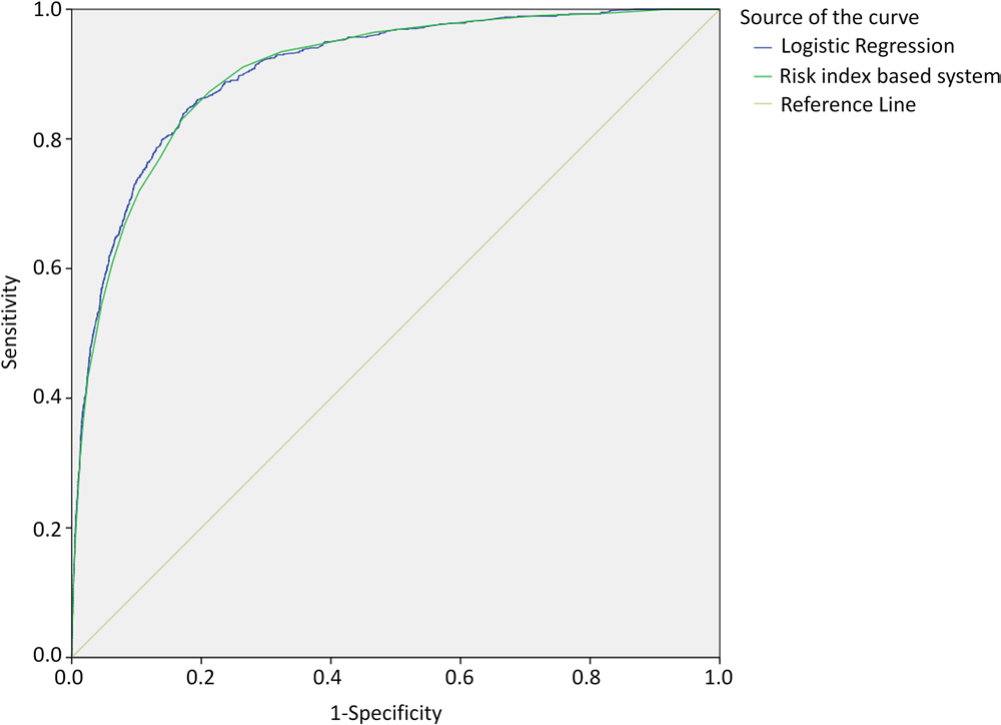
The ROC curves for predicting nosocomial lower respiratory tract infections derived from logistic regression and risk index based system.

**Figure 4:**
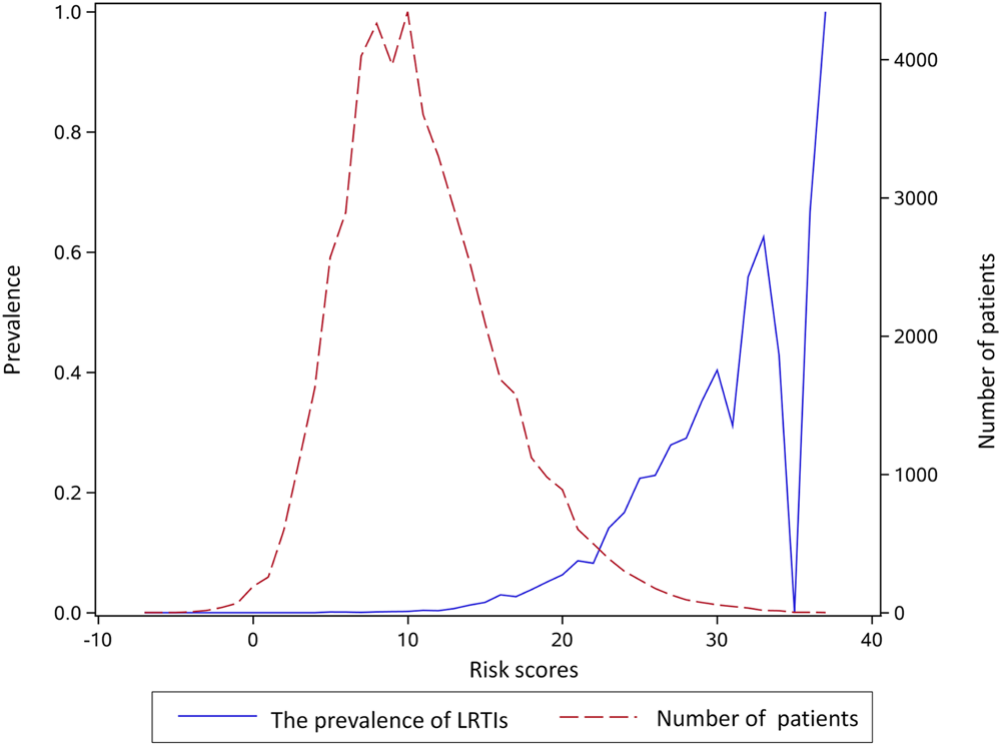
The number of patients and prevalence of nosocomial lower respiratory tract infections among patients with different risk scores.

As Fig. 2 reveals, the area under ROC curve was 0.907 for internal validation, and 0.888 for 10-fold cross validation. The sensitivity and specificity of the best cut-off point that logistic regression internal validation can achieve was 0.86 and 0.79, respectively, and that of 10-fold cross validation is 0.87 and 0.74, respectively. The logistic model was stable, so we chose logistic regression coefficient to construct the risk index based system for predicting nosocomial LRTIs. The system includes ten categories of risk factors, each corresponds to a special risk score (Table 1). The overall risk score for each patient is the sum of the risk scores for all the potential risk factors of this patient (Table 1). The ROC curves of logistic regression and risk index scoring system are very similar (Fig. 3), indicating no significant loss of accuracy. The area under the ROC curve for the risk index system was 0.905 (95% CI, 0.895 to 0.915). As risk score increases, the prevalence of LRTIs increases sharply (Fig. 4). For most of the patients, the total risk score represents the possibility of acquiring nosocomial LRTIs. Six risk levels were generated according to the total risk scores of all the study population, ranging from 0 to 5, the corresponding prevalence of nosocomial LRTIs were 0.00%, 0.39%, 3.86%, 12.38%, 28.79% and 44.83%, respectively (Table 2).

**Table 1:** The scoring system based on risk factors of nosocomial lower respiratory tract infections.

Risk factor variables	Risk scores for each variable (points)
Presence of one of the following diseases: Enlarged prostate (N40), Malignant neoplasm of colon (C18)	−8
Use of antibiotics for prophylactic purpose, Type IV incision operation	−3
Type I incision operation, Age (5 points for patients of <4 years, 0 points for patients of 4–9 years, 1 points per 10 years for patients of ≥10 years)	1
Length of hospital stay (2 points per week until the total score reach 10 points), Mechanical ventilatory support in place, Presence of one of the following diseases: Cerebral infarction (I63), Intracranial injury (S06), Sequelae of cerebrovascular disease (I69)	2
Tracheotomy, Central or peripheral catheter in place, Male, Presence of one of the following diseases: Acute bronchitis (J20), Emphysema (J43), Encounter for follow-up examination after completed treatment for malignant neoplasm (Z08), Intracranial and intraspinal abscess and granuloma (G06), Malignant neoplasm of esophagus (C15), Malignant neoplasm of liver and intrahepatic bile ducts (C22), Other disorders of brain (G93), Other pleural conditions (J94), Other diseases of digestive system (K92), Secondary malignant neoplasm of respiratory and digestive organs (C78), Unspecified kidney failure (N19)	3
Urinary catheter in place, Presence of one of the following diseases: Acute pancreatitis (K85), Malignant neoplasm of bronchus and lung (C34), Malignant neoplasm of brain (C71), Malignant neoplasm without specification of site (C80), Nontraumatic intracerebral hemorrhage (I61), Other sepsis (A41), Other degenerative diseases of nervous system, not elsewhere classified (G31), Transplanted organ and tissue status (Z94)	4
Presence of one of the following diseases: Acute myocardial infarction (I21), Acute tonsillitis (J03), Complications and ill-defined descriptions of heart disease (I51), Hepatic failure, not elsewhere classified (K72), Intraoperative and postprocedural complications and disorders of digestive system, not elsewhere classified (K91), Other and unspecified types of non-Hodgkin lymphoma (C85), Other rheumatoid arthritis (M06), Pneumothorax and air leak (J93), Rheumatic mitral valve diseases (I05)	5
Presence of one of the following diseases: Acute nephritic syndrome (N00), Diaphragmatic hernia (K44), Esophageal varices (I85), Leukemia of unspecified cell type (C95), leukemias of specified cell type (C94), Lymphoid leukemia (C91), Myeloid leukemia (C92), Neoplasm of uncertain or unknown behaviour of oral cavity and digestive organs (D37), Open wound of head (S01), Other aplastic anaemias (D61), Other congenital malformations of circulatory system (Q28), Other Retention of urine (R33), Respiratory conditions due to other external agents (J70), Systemic lupus erythematosus (M32)	8
Presence of one of the following diseases: Arterial embolism and thrombosis (I74), Congenital pneumonia (P23), Malignant neoplasm of floor of mouth (C04), Other birth injuries (P15), Malignant neoplasm of other and unspecified female genital organs (C57), Occlusion and stenosis of cerebral arteries, not resulting in cerebral infarction (I66), Other disorders of cartilage (M94), Pain in throat and chest (R07), Toxic effect of pesticides (T60)	10
Presence of one of the following diseases: Excessive, frequent and irregular menstruation (N92), Other conditions originating in the perinatal period (P96), Other lack of coordination (R27), Other specified diseases with participation of lymphoreticular and reticulohistiocytic tissue (D76)	15

The overall risk score for each patient is the sum of the scores for all the risk factor variables. The presence of underlying diseases was shown as disease type and its corresponding clinical modification (ICD-10-CM) code.

**Table 2:** The prevalence of lower respiratory tract infections among patients with different risk levels.

Risk levels	Risk score range	No. of susceptible patients	No. of LRTI cases	Prevalence of LRTIs (%)
0	−7 to 4	3901	0	0.00%
1	5 to 15	36507	143	0.39%
2	16 to 20	6249	241	3.86%
3	21 to 25	2027	251	12.38%
4	26 to 30	528	152	28.79%
5	31 to 37	116	52	44.83%
total	−7 to 37	49328	839	1.70

All the potential cut-off points are shown in Table 3. Both the sensitivity and specificity were higher than 0.70, while the positive likelihood ratio and negative likelihood ratio varied from 3.50 to 7.20, and from 0.12 to 0.31, respectively. In practical application, one can choose the best cut-off point based on the specific task. In this study, we suggest 14 as the best cut-off point, for which the sensitivity and specificity were 0.87 and 0.79, respectively. To assess the predictive ability of 14, we calculated the sensitivity and specificity in subgroups with different diagnosis. The best cut-off point showed high discriminatory power in the majority of the subgroups with different underlying diseases, such as respiratory tuberculosis, malignant neoplasm of nasopharynx, malignant neoplasm of stomach, unspecified diabetes mellitus, *et al*. (Supplemental Table S3).

**Table 3:** The performance of predication for lower respiratory tract infections using different cut-off values.

Cut-off values	No. of true positive cases	No. of false positive cases	No. of true negative cases	No. of false negative cases	Sensitivity	Specificity	Positive likelihood ratio	Negative likelihood ratio
13	764	12791	35698	75	0.91	0.74	3.50	0.12
14	732	10284	38205	107	0.87	0.79	4.14	0.16
15	696	8224	40265	143	0.83	0.83	4.88	0.20
16	646	6589	41900	193	0.77	0.86	5.50	0.27
17	604	5056	43433	235	0.72	0.90	7.20	0.31

## Discussions

Predication of LRTIs among non-ventilated patients is very important for the early implementation of prevention strategies, including oral care, early mobilization interventions, swift diagnosis and treatment of dysphagia, as well as antimicrobial prophylaxis^19,20^. This study developed a risk index based system for LRTIs, which assigns a corresponding risk score to each patient according to the presence of risk factors, e.g. underlying diseases and medical treatment. The total scores derived from the risk factors of one patient would represent his/her risk for acquiring LRTI. Targeted prevention and control strategies could be implemented by healthcare workers according to the results of risk evaluation, and the cost-effective of infection control programs in hospitals is expected to be improved.

In general, the discriminatory power of two statistical models and the risk index based system in predicting nosocomial LRTIs was excellent and comparable with previous studies^9,15,21^. The overall prevalence of LRTIs in this study was 1.7%, while for the patients with risk level at four and five, the prevalence were as high as around 30% and 40%, respectively, which indicates that the system and the recommended best cut-off point could be used to identify patients with high risk. Active preventive measures could be taken for these patients^19,22,23^.

As the susceptibility of LRTIs among patients with different underlying diseases might be different, it is reasonable that each patient’s underlying diseases should be treated as important risk factors and be included for constructing the risk index model. There was a fluctuation for the number of LRTI patients when the total risk score was above 30 (Fig. 3), which was possibly associated with the reduction of number of susceptible patients. For some patients with certain underlying diseases, the predicative effectiveness was suboptimal possibly due to lack of information on patients’ physiology status.

This study has some limitations. Firstly, no external validation was conducted in this study, the applicability of this risk predicative system in clinical field deserves further study; Secondly, the data of this study comes from a point-prevalence survey, no follow up was conducted for the study population, some patients, who acquired LRTI after the survey date, might be misclassified as non-LRTI cases; Thirdly, the missing of some risk factor data might affect the accuracy of model construction. However, the high sensitivity and specificity of the system in this study suggested that it might be applied to boost more rational and cost-effective infection control programs for nosocomial LRTIs.

## Electronic supplementary material

Supplemental file

## References

[1] Magill SS (2014). Multistate point-prevalence survey of health care-associated infections. N Engl J Med.

[2] Huttner A (2013). Antimicrobial resistance: a global view from the 2013 World Healthcare-Associated Infections Forum. Antimicrob Resist Infect Control.

[3] Marcel JP (2008). Healthcare-associated infections: think globally, act locally. Clin Microbiol Infect.

[4] Alp E, Damani N (2015). Healthcare-associated infections in intensive care units: epidemiology and infection control in low-to-middle income countries. J Infect Dev Ctries.

[5] Zarb, P. *et al*. The European Centre for Disease Prevention and Control (ECDC) pilot point prevalence survey of healthcare-associated infections and antimicrobial use. *Euro Surveill* **17** (2012). 10.2807/ese.17.46.20316-en 23171822

[6] Li C (2014). Point-prevalence of healthcare-associated infection in china in 2010: a large multicenter epidemiological survey. Infect Control Hosp Epidemiol.

[7] Olsen MA (2016). Development of a Risk Prediction Model to Individualize Risk Factors for Surgical Site Infection After Mastectomy. Ann Surg Oncol.

[8] Fukuda H, Kuroki M (2016). The Development of Statistical Models for Predicting Surgical Site Infections in Japan: Toward a Statistical Model-Based Standardized Infection Ratio. Infect Control Hosp Epidemiol.

[9] Saptharishi LG, Jayashree M, Singhi S (2016). Development and validation of the “Pediatric Risk of Nosocomial Sepsis (PRiNS)” score for health care-associated infections in a medical pediatric intensive care unit of a developing economy–a prospective observational cohort study. J Crit Care.

[10] Celis R (1988). Nosocomial pneumonia. A multivariate analysis of risk and prognosis. Chest.

[11] Drakulovic MB (1999). Supine body position as a risk factor for nosocomial pneumonia in mechanically ventilated patients: a randomised trial. Lancet.

[12] Wolkewitz M (2008). Risk factors for the development of nosocomial pneumonia and mortality on intensive care units: application of competing risks models. Crit Care.

[13] Gastmeier P, Menzel K, Sohr D, Ruden H (2007). Usefulness of severity-of-illness scores based on admission data only in nosocomial infection surveillance systems. Infect Control Hosp Epidemiol.

[14] Strand K, Flaatten H (2008). Severity scoring in the ICU: a review. Acta Anaesthesiol Scand.

[15] Chang YJ (2011). Predicting hospital-acquired infections by scoring system with simple parameters. PLoS ONE.

[16] Paryavi E (2013). Predictive model for surgical site infection risk after surgery for high-energy lower-extremity fractures: development of the risk of infection in orthopedic trauma surgery score. J Trauma Acute Care Surg.

[17] Chen Y (2017). A point-prevalence survey of healthcare-associated infection in fifty-two Chinese hospitals. J Hosp Infect.

[18] Dreiseitl S, Ohno-Machado L (2002). Logistic regression and artificial neural network classification models: a methodology review. J Biomed Inform.

[19] Passaro L, Harbarth S, Landelle C (2016). Prevention of hospital-acquired pneumonia in non-ventilated adult patients: a narrative review. Antimicrob Resist Infect Control.

[20] Lesho E (2005). Role of inhaled antibacterials in hospital-acquired and ventilator-associated pneumonia. Expert Rev Anti Infect Ther.

[21] Velasco E, Thuler LC, Martins CA, Dias LM, Goncalves VM (1998). Risk index for prediction of surgical site infection after oncology operations. Am J Infect Control.

[22] Panchabhai TS, Dangayach NS, Krishnan A, Kothari VM, Karnad DR (2009). Oropharyngeal cleansing with 0.2% chlorhexidine for prevention of nosocomial pneumonia in critically ill patients: an open-label randomized trial with 0.01% potassium permanganate as control. Chest.

[23] Craven DE, Steger KA, Barat LM, Duncan RA (1992). Nosocomial pneumonia: epidemiology and infection control. Intensive Care Med.

