# Supplementary material for: Predicting nosocomial lower respiratory tract infections by a risk index based system
Source: Sci Rep. 2017 Nov 21;7:15933. doi: 10.1038/s41598-017-15765-z (PMC5698311; doi:10.1038/s41598-017-15765-z)
Supplement: Supplementary file 1 — Supplemental file [file 41598_2017_15765_MOESM1_ESM.pdf]

Electronic Supporting Information

Predicting nosocomial lower respiratory tract infections by a risk index based system

Yong Chen[1#], Xue Shan[2#], Jingya Zhao[1], Xuelin Han[1], Shuguang Tian[1], Fangyan Chen[1], Xueting Su[1], Yansong Sun[1], Liuyu Huang[1], Hajo Grundmann[3,4], Hongyuan Wang[2*], Li Han[1*]

1 Chinese PLA Institute for Disease Control and Prevention, Beijing, China,

2 School of Public Health, Peking University, Beijing, China

3 Department of Infection Prevention and Hospital Hygiene, Faculty of Medicine, University of Freiburg, Freiburg, Germany.

4 Department of Medical Microbiology, University Medical Center Groningen, Rijksuniversteit Groningen, Groningen, The Netherlands.

Running title: Predicting nosocomial LRTIs by simplified index

[#]These authors contribute equally to this work.

***Corresponding author:**

Dr. Li Han; Mailing address: Chinese PLA Institute for Disease Control & Prevention, 20# Dongda Str., 100071, Beijing, China. E-mail: hanlicdc@163.com.

Dr. Hongyuan Wang; Mailing address: School of Public Health, Peking University, 38# Xueyuan Road, 100191, Beijing, China. E-mail:why_w2003@163.com.

Table S1.Variables used for statistical analysis

Variables	Assignment
Age (decades)	Continuous variable from 0 to 10, larger than 10 were set to 10
Gender	0-Male, 1-Female
Length of hospital stay (weeks)	Continuous variable from 0 to 4, lager than 4 were set to 4
Central or peripheral catheter in place	0- No, 1-Yes
Urinary catheter in place	0- No, 1-Yes
Mechanical ventilatory support in place	0- No, 1-Yes
Hemodialysis	0- No, 1-Yes
Surgery	0-No,1-type 1 incision,2-type 2 incision,3-type 3incision, 4-type 4incision
Tracheotomy	0- No, 1-Yes
Use of antibiotics for prophylactic purpose	0- No, 1-Yes
Underlying disease (each of ICD-10-CM three character categories as a binary variable)	0- No, 1-Yes

Table S2. The prevalence of lower respiratory tract infections (LRTIs) among patients with different underlying disease.

ICD-10-CM code	Diagnosis of disease	Number of susceptible patients	Number of patients with LRTI	Prevalence of LRTIs (%)
A09	Infectious gastroenteritis and colitis, unspecified	169	4	2.4
A15	Respiratory tuberculosis	193	3	1.6
A41	Other sepsis	75	8	10.7
A46	Erysipelas	23	1	4.3
B16	Acute hepatitis B	412	4	1
B18	Chronic viral hepatitis	214	2	0.9
B25	Cytomegaloviral disease	10	1	10
B90	Sequelae of tuberculosis	35	1	2.9
C02	Malignant neoplasm of other and unspecified parts of tongue	18	1	5.6
C04	Malignant neoplasm of floor of mouth	14	2	14.3
C11	Malignant neoplasm of nasopharynx	399	3	0.8
C13	Malignant neoplasm of hypopharynx	41	2	4.9
C15	Malignant neoplasm of esophagus	519	19	3.7
C16	Malignant neoplasm of stomach	629	14	2.2
C18	Malignant neoplasm of colon	398	1	0.3
C20	Malignant neoplasm of rectum	216	1	0.5
C22	Malignant neoplasm of liver and intrahepatic bile ducts	620	14	2.3
C25	Malignant neoplasm of pancreas	170	3	1.8
C26	Malignant neoplasm of other and ill-defined digestive organs	25	2	8
C32	Malignant neoplasm of larynx	79	3	3.8
C34	Malignant neoplasm of bronchus and lung	1505	48	3.2
C37	Malignant neoplasm of	24	1	4.2

	thymus			
C41	Malignant neoplasm of bone and articular cartilage of other and unspecified sites	24	1	4.2
C48	Malignant neoplasm of retroperitoneum and peritoneum	10	1	10
C50	Malignant neoplasm of breast	762	2	0.3
C53	Malignant neoplasm of cervix uteri	260	1	0.4
C56	Malignant neoplasm of ovary	157	1	0.6
C57	Malignant neoplasm of other and unsp female genital organs	4	1	25
C61	Malignant neoplasm of prostate	131	7	5.3
C64	Malignant neoplasm of kidney, except renal pelvis	93	4	4.3
C66	Malignant neoplasm of ureter	16	1	6.3
C67	Malignant neoplasm of bladder	115	1	0.9
C71	Malignant neoplasm of brain	80	4	5
C73	Malignant neoplasm of thyroid gland	154	1	0.6
C76	Malignant neoplasm of other and ill-defined sites	12	1	8.3
C78	Secondary malignant neoplasm of respiratory and digestive organs	348	11	3.2
C79	Secondary malignant neoplasm of other and unspecified sites	296	8	2.7
C80	Malignant neoplasm without specification of site	195	10	5.1
C83	Non-follicular lymphoma	53	1	1.9
C84	Mature T/NK-cell lymphomas	9	1	11.1
C85	Other and unspecified types of non-Hodgkin lymphoma	259	11	4.2
C90	Multiple myeloma and malignant plasma cell neoplasms	116	3	2.6
C91	Lymphoid leukemia	227	12	5.3

C92	Myeloid leukemia	235	13	5.5
C94	Other leukemias of specified cell type	28	2	7.1
C95	Leukemia of unspecified cell type	172	7	4.1
D32	Benign neoplasm of meninges	104	5	4.8
D37	Neoplasm of uncertain or unknown behaviour of oral cavity and digestive organs	9	1	11.1
D46	Myelodysplastic syndromes	75	4	5.3
D47	Other neoplasms of uncertain or unknown behaviour of lymphoid, haematopoietic and related tissue	12	1	8.3
D50	Iron deficiency anemia	35	1	2.9
D61	Other aplastic anaemias	86	4	4.7
D64	Other anemias	469	8	1.7
D69	Purpura and other hemorrhagic conditions	130	2	1.5
D73	Diseases of spleen	18	1	5.6
D75	Other diseases of blood and blood-forming organs	19	1	5.3
D76	Other specified diseases with participation of lymphoreticular and reticulohistiocytic tissue	3	1	33.3
E03	Other hypothyroidism	74	2	2.7
E04	Other nontoxic goiter	94	1	1.1
E05	Thyrotoxicosis [hyperthyroidism]	95	1	1.1
E10	Type 1 diabetes mellitus	48	1	2.1
E11	Type 2 diabetes mellitus	2048	28	1.4
E14	Unspecified diabetes mellitus	1408	19	1.3
E43	Unspecified severe protein-calorie malnutrition	7	1	14.3
E46	Unspecified protein-calorie malnutrition	10	1	10
E77	Disorders of glycoprotein metabolism	91	6	6.6
E78	Disorders of lipoprotein metabolism and other lipidemias	469	1	0.2

E87	Other disorders of fluid, electrolyte and acid-base balance	167	3	1.8
F01	Vascular dementia	22	3	13.6
F03	Unspecified dementia	53	5	9.4
F06	Other mental disorders due to known physiological condition	10	1	10
G04	Encephalitis, myelitis and encephalomyelitis	40	1	2.5
G06	Intracranial and intraspinal abscess and granuloma	73	9	12.3
G12	Spinal muscular atrophy and related syndromes	23	1	4.3
G20	Parkinson's disease	144	9	6.3
G30	Alzheimer's disease	58	5	8.6
G31	Other degenerative diseases of nervous system, not elsewhere classified	63	6	9.5
G35	Multiple sclerosis	11	1	9.1
G40	Epilepsy and recurrent seizures	294	13	4.4
G45	Transient cerebral ischemic attacks and related syndromes	204	5	2.5
G47	Sleep disorders	55	1	1.8
G50	Disorders of trigeminal nerve	19	1	5.3
G61	Inflammatory polyneuropathy	15	1	6.7
G70	Myasthenia gravis and other myoneural disorders	33	1	3
G82	Paraplegia (paraparesis) and quadriplegia (quadriparesis)	118	6	5.1
G91	Hydrocephalus	119	13	10.9
G93	Other disorders of brain	183	18	9.8
G95	Other and unspecified diseases of spinal cord	43	1	2.3
I05	Rheumatic mitral valve diseases	62	5	8.1
I09	Other rheumatic heart diseases	171	10	5.8
I10	Essential (primary) hypertension	5466	113	2.1
I15	Secondary hypertension	106	1	0.9
I20	Angina pectoris	245	4	1.6
I21	Acute myocardial infarction	140	5	3.6

I25	Chronic ischemic heart disease	3971	97	2.4
I26	Pulmonary embolism	37	1	2.7
I27	Other pulmonary heart diseases	149	6	4
I35	Nonrheumatic aortic valve disorders	41	1	2.4
I38	Endocarditis, valve unspecified	29	1	3.4
I46	Cardiac arrest	23	3	13
I47	Paroxysmal tachycardia	91	1	1.1
I48	Atrial fibrillation and flutter	184	8	4.3
I49	Other cardiac arrhythmias	308	5	1.6
I50	Heart failure	411	15	3.6
I51	Complications and ill-defined descriptions of heart disease	157	4	2.5
I60	Nontraumatic subarachnoid hemorrhage	194	13	6.7
I61	Nontraumatic intracerebral hemorrhage	670	69	10.3
I62	Other and unspecified nontraumatic intracranial hemorrhage	114	3	2.6
I63	Cerebral infarction	2339	75	3.2
I65	Occlusion and stenosis of precerebral arteries, not resulting in cerebral infarction	67	2	3
I66	Occlusion and stenosis of cerebral arteries, not resulting in cerebral infarction	42	7	16.7
I67	Other cerebrovascular diseases	885	21	2.4
I69	Sequelae of cerebrovascular disease	656	39	5.9
I70	Atherosclerosis	169	1	0.6
I71	Aortic aneurysm and dissection	64	3	4.7
I72	Other aneurysm	10	1	10
I74	Arterial embolism and thrombosis	3	1	33.3
I82	Other venous embolism and thrombosis	21	1	4.8
I85	Esophageal varices	11	1	9.1
I89	Other noninfective disorders	3	1	33.3

	of lymphatic vessels and lymph nodes			
J06	Acute upper respiratory infections of multiple and unspecified sites	464	4	0.9
J20	Acute bronchitis	514	9	1.8
J31	Chronic rhinitis, nasopharyngitis and pharyngitis	106	1	0.9
J33	Nasal polyp	45	1	2.2
J34	Other and unspecified disorders of nose and nasal sinuses	118	1	0.8
J38	Diseases of vocal cords and larynx, not elsewhere classified	76	1	1.3
J42	Unspecified chronic bronchitis	286	14	4.9
J43	Emphysema	156	9	5.8
J44	Other chronic obstructive pulmonary disease	523	23	4.4
J45	Asthma	179	2	1.1
J47	Bronchiectasis	152	5	3.3
J69	Pneumonitis due to solids and liquids	112	10	8.9
J70	Respiratory conditions due to other external agents	6	1	16.7
J80	Acute respiratory distress syndrome	14	1	7.1
J84	Other interstitial pulmonary diseases	48	3	6.3
J85	Abscess of lung and mediastinum	22	1	4.5
J86	Pyothorax	19	2	10.5
J93	Pneumothorax and air leak	66	4	6.1
J94	Other pleural conditions	281	14	5
J96	Respiratory failure, not elsewhere classified	243	27	11.1
J98	Other respiratory disorders	1418	246	17.3
K05	Gingivitis and periodontal diseases	6	2	33.3
K21	Gastro-esophageal reflux disease	114	3	2.6

K22	Other diseases of esophagus	22	1	4.5
K25	Gastric ulcer	84	1	1.2
K26	Duodenal ulcer	136	1	0.7
K29	Gastritis and duodenitis	650	4	0.6
K31	Other diseases of stomach and duodenum	90	1	1.1
K44	Diaphragmatic hernia	15	2	13.3
K52	Other noninfective gastroenteritis and colitis	45	1	2.2
K56	Paralytic ileus and intestinal obstruction without hernia	228	4	1.8
K60	Fissure and fistula of anal and rectal regions	121	1	0.8
K65	Peritonitis	148	4	2.7
K70	Alcoholic liver disease	64	2	3.1
K71	Toxic liver disease	56	1	1.8
K72	Hepatic failure, not elsewhere classified	40	2	5
K74	Fibrosis and cirrhosis of liver	709	12	1.7
K76	Other diseases of liver	381	3	0.8
K80	Cholelithiasis	712	7	1
K81	Cholecystitis	235	1	0.4
K83	Other diseases of biliary tract	105	1	1
K85	Acute pancreatitis	186	7	3.8
K86	Other diseases of pancreas	49	1	2
K91	Postprocedural disorders of digestive system, not elsewhere classified	80	6	7.5
K92	Other diseases of digestive system	346	12	3.5
L08	Other local infections of skin and subcutaneous tissue	38	1	2.6
L40	Psoriasis	130	1	0.8
L80	Vitiligo	18	1	5.6
L89	Pressure ulcer	57	3	5.3
L97	Non-pressure chronic ulcer of lower limb, NEC	5	1	20
M06	Other rheumatoid arthritis	183	5	2.7
M13	Other arthritis	193	1	0.5
M19	Other and unspecified osteoarthritis	3	1	33.3

M32	Systemic lupus erythematosus (SLE)	114	2	1.8
M35	Other systemic involvement of connective tissue	45	1	2.2
M41	Scoliosis	59	1	1.7
M45	Ankylosing spondylitis	89	1	1.1
M47	Spondylosis	429	2	0.5
M50	Cervical disc disorders	449	1	0.2
M94	Other disorders of cartilage	4	1	25
N00	Acute nephritic syndrome	14	1	7.1
N04	Nephrotic syndrome	258	4	1.6
N17	Acute kidney failure	22	1	4.5
N18	Chronic kidney disease (CKD)	906	17	1.9
N19	Unspecified kidney failure	483	19	3.9
N20	Calculus of kidney and ureter	283	2	0.7
N39	Other disorders of urinary system	506	15	3
N40	Enlarged prostate	395	1	0.3
N92	Excessive, frequent and irregular menstruation	3	1	33.3
P07	Disorders related to short gestation and low birth weight, not elsewhere classified	192	5	2.6
P15	Other birth injuries	7	1	14.3
P21	Birth asphyxia	46	2	4.3
P23	Congenital pneumonia	306	12	3.9
P29	Cardiovascular disorders originating in the perinatal period	10	1	10
P59	Neonatal jaundice from other and unspecified causes	148	1	0.7
P96	Other conditions originating in the perinatal period	3	1	33.3
Q21	Congenital malformations of cardiac septa	97	2	2.1
Q24	Other congenital malformations of heart	158	2	1.3
Q28	Other congenital malformations of circulatory system	9	1	11.1
R02	Gangrene, not elsewhere classified	14	1	7.1

R07	Pain in throat and chest	6	1	16.7
R09	Other symptoms and signs involving the circulatory and respiratory systems	8	1	12.5
R18	Ascites	67	2	3
R27	Other lack of coordination	8	2	25
R31	Hematuria	40	1	2.5
R33	Retention of urine	47	3	6.4
R40	Somnolence, stupor and coma	2	1	50
R57	Shock, not elsewhere classified	91	10	11
R68	Other general symptoms and signs	20	5	25
R90	Abnormal findings on diagnostic imaging of central nervous system	55	2	3.6
R91	Abnormal findings on diagnostic imaging of lung	131	1	0.8
S01	Open wound of head	22	2	9.1
S02	Fracture of skull and facial bones	219	7	3.2
S06	Intracranial injury	776	49	6.3
S09	Other and unspecified injuries of head	117	1	0.9
S12	Fracture of cervical vertebra and other parts of neck	72	3	4.2
S14	Injury of nerves and spinal cord at neck level	49	1	2
S22	Fracture of rib(s), sternum and thoracic spine	265	1	0.4
S27	Injury of other and unspecified intrathoracic organs	90	5	5.6
S29	Other and unspecified injuries of thorax	46	1	2.2
S32	Fracture of lumbar spine and pelvis	459	5	1.1
S39	Other and unspecified injuries of abdomen, lower back and pelvis	38	1	2.6
S72	Fracture of femur	681	8	1.2
S82	Fracture of lower leg, including ankle	703	3	0.4

T02	Fractures involving multiple body regions	68	5	7.4
T07	Unspecified multiple injuries	160	3	1.9
T09	Other injuries of spine and trunk, level unspecified	35	1	2.9
T29	Burns and corrosions of multiple body regions	209	3	1.4
T60	Toxic effect of pesticides	4	1	25
T79	Certain early complications of trauma, NEC	8	1	12.5
T86	Complications of transplanted organs and tissue	65	3	4.6
T90	Sequelae of injuries of head	65	2	3.1
Z08	Encntr for follow-up exam after trtmt for malignant neoplasm	218	7	3.2
Z93	Artificial opening status	22	2	9.1
Z94	Transplanted organ and tissue status	281	16	5.7
Z95	Presence of cardiac and vascular implants and grafts	54	1	1.9
Z98	Other postprocedural states	52	2	3.8

Table S3. The performance of the best cut-off point in predicting nosocomial lower respiratory tract infections for patients with different underlying diseases.

ICD10CM code	No. of true positive cases	No. of false negative cases	No. of false positive cases	No. of true negative cases	No. of patients	The prevalence of LRTI	Sensitivity	Specificity	Diagnosis of disease
A09	3	1	19	135	158	0.03	0.75	0.88	
A15	3	0	29	157	189	0.02	1	0.84	Respiratory tuberculosis
A15	3	0	28	158	189	0.02	1	0.85	Respiratory tuberculosis
A41	8	0	40	24	72	0.11	1	0.38	Other sepsis
A41	8	0	40	24	72	0.11	1	0.38	Other sepsis
A46	1	0	6	15	22	0.05	1	0.71	Erysipelas
A46	1	0	5	16	22	0.05	1	0.76	Erysipelas
A49	1	0	6	37	44	0.02	1	0.86	Bacterial infection of unspecified site
A49	1	0	6	37	44	0.02	1	0.86	Bacterial infection of unspecified site
B16	2	2	51	349	404	0.01	0.5	0.87	Acute hepatitis B
B16	2	2	53	347	404	0.01	0.5	0.87	Acute hepatitis B
B18	1	1	23	174	199	0.01	0.5	0.88	Chronic viral hepatitis
B18	1	1	23	174	199	0.01	0.5	0.88	Chronic viral hepatitis
B25	1	0	2	7	10	0.1	1	0.78	Cytomegaloviral disease
B25	1	0	1	8	10	0.1	1	0.89	Cytomegaloviral disease
B90	0	1	11	21	33	0.03	0	0.66	Sequelae of tuberculosis
B90	0	1	11	21	33	0.03	0	0.66	Sequelae of tuberculosis
C02	1	0	3	14	18	0.06	1	0.82	Malignant neoplasm of other and unspecified parts of tongue
C04	2	0	11	0	13	0.15	1	0	Malignant neoplasm of floor of mouth
C11	3	0	59	314	376	0.01	1	0.84	Malignant neoplasm of nasopharynx
C13	2	0	10	24	36	0.06	1	0.71	Malignant neoplasm of hypopharynx
C15	18	1	223	243	485	0.04	0.95	0.52	Malignant neoplasm of esophagus
C16	12	2	118	467	599	0.02	0.86	0.8	Malignant neoplasm of stomach
C18	0	1	11	358	370	0	0	0.97	Malignant neoplasm of colon
C20	1	0	75	124	200	0	1	0.62	Malignant neoplasm of rectum

C22	11	3	211	358	583	0.02	0.79	0.63	Malignant neoplasm of liver and intrahepatic bile ducts
C25	1	2	44	114	161	0.02	0.33	0.72	Malignant neoplasm of pancreas
C26	2	0	6	15	23	0.09	1	0.71	Malignant neoplasm of other and ill-defined digestive organs
C32	2	1	22	53	78	0.04	0.67	0.71	Malignant neoplasm of larynx
C34	46	2	750	605	1403	0.03	0.96	0.45	Malignant neoplasm of bronchus and lung
C37	0	1	3	17	21	0.05	0	0.85	Malignant neoplasm of thymus
C41	1	0	2	19	22	0.05	1	0.9	Malignant neoplasm of bone/artic cartl of and unsp sites
C48	1	0	2	6	9	0.11	1	0.75	Malignant neoplasm of retroperitoneum and peritoneum
C50	0	2	52	623	677	0	0	0.92	Malignant neoplasm of breast
C53	0	1	8	238	247	0	0	0.97	Malignant neoplasm of cervix uteri
C56	1	0	11	136	148	0.01	1	0.93	Malignant neoplasm of ovary
C57	1	0	3	0	4	0.25	1	0	Malignant neoplasm of other and unsp female genital organs
C61	7	0	44	72	123	0.06	1	0.62	Malignant neoplasm of prostate
C64	4	0	17	63	84	0.05	1	0.79	Malignant neoplasm of kidney, except renal pelvis
C66	1	0	5	8	14	0.07	1	0.62	Malignant neoplasm of ureter
C67	1	0	38	72	111	0.01	1	0.65	Malignant neoplasm of bladder
C71	4	0	37	37	78	0.05	1	0.5	Malignant neoplasm of brain
C73	1	0	12	130	143	0.01	1	0.92	Malignant neoplasm of thyroid gland
C76	1	0	2	9	12	0.08	1	0.82	Malignant neoplasm of other and ill-defined sites
C78	8	3	134	178	323	0.03	0.73	0.57	Secondary malignant neoplasm of resp and digestive organs
C79	6	2	75	200	283	0.03	0.75	0.73	Secondary malignant neoplasm of other and unspecified sites

C80	7	3	105	74	189	0.05	0.7	0.41	Malignant neoplasm without specification of site
C83	0	1	15	35	51	0.02	0	0.7	Non-follicular lymphoma
C84	1	0	1	7	9	0.11	1	0.88	Mature T/NK-cell lymphomas
C85	11	0	110	129	250	0.04	1	0.54	Oth and unspecified types of non-Hodgkin lymphoma
C90	1	2	18	85	106	0.03	0.33	0.83	Multiple myeloma and malignant plasma cell neoplasms
C91	11	1	158	54	224	0.05	0.92	0.25	Lymphoid leukemia
C92	13	0	185	32	230	0.06	1	0.15	Myeloid leukemia
C94	2	0	21	3	26	0.08	1	0.13	Other leukemias of specified cell type
C95	7	0	130	24	161	0.04	1	0.16	Leukemia of unspecified cell type
D32	5	0	39	52	96	0.05	1	0.57	Benign neoplasm of meninges
D37	1	0	7	0	8	0.13	1	0	Neoplasm of uncrt behavior of oral cavity and dgstv organs
D46	3	1	22	46	72	0.06	0.75	0.68	Myelodysplastic syndromes
D47	0	1	2	6	9	0.11	0	0.75	Oth neoplm of uncrt behav of lymphoid, hematpoetc & rel tiss
D50	0	1	2	31	34	0.03	0	0.94	Iron deficiency anemia
D61	3	1	60	19	83	0.05	0.75	0.24	Oth aplastic anemias and other bone marrow failure syndromes
D64	7	1	88	332	428	0.02	0.88	0.79	Other anemias
D69	0	2	9	109	120	0.02	0	0.92	Purpura and other hemorrhagic conditions
D73	1	0	1	14	16	0.06	1	0.93	Diseases of spleen
D75	1	0	5	13	19	0.05	1	0.72	Other and unsp diseases of blood and blood-forming organs
D76	1	0	2	0	3	0.33	1	0	Oth dis with lymphoreticular and reticulohistiocytic tissue
E03	2	0	12	53	67	0.03	1	0.82	Other hypothyroidism
E04	1	0	7	79	87	0.01	1	0.92	Other nontoxic

									goiter
E05	0	1	4	84	89	0.01	0	0.95	Thyrotoxicosis [hyperthyroidism]
E10	0	1	6	38	45	0.02	0	0.86	Type 1 diabetes mellitus
E11	22	6	448	1460	1936	0.01	0.79	0.77	Type 2 diabetes mellitus
E14	16	3	265	1039	1323	0.01	0.84	0.8	Unspecified diabetes mellitus
E43	1	0	3	3	7	0.14	1	0.5	Unspecified severe protein-calorie malnutrition
E46	1	0	3	6	10	0.1	1	0.67	Unspecified protein-calorie malnutrition
E77	6	0	31	48	85	0.07	1	0.61	Disorders of glycoprotein metabolism
E78	0	1	48	393	442	0	0	0.89	Disorders of lipoprotein metabolism and other lipidemias
E87	3	0	46	106	155	0.02	1	0.7	Other disorders of fluid, electrolyte and acid-base balance
F01	3	0	9	10	22	0.14	1	0.53	Vascular dementia
F03	5	0	30	17	52	0.1	1	0.36	Unspecified dementia
F06	1	0	3	5	9	0.11	1	0.63	Other mental disorders due to known physiological condition
G04	1	0	7	31	39	0.03	1	0.82	Encephalitis, myelitis and encephalomyelitis
G06	9	0	38	22	69	0.13	1	0.37	Intracranial and intraspinal abscess and granuloma
G12	1	0	10	11	22	0.05	1	0.52	Spinal muscular atrophy and related syndromes
G20	9	0	67	55	131	0.07	1	0.45	Parkinson's disease
G30	5	0	29	20	54	0.09	1	0.41	Alzheimer's disease
G31	6	0	45	10	61	0.1	1	0.18	Other degenerative diseases of nervous system, not elsewhere classified
G35	1	0	0	10	11	0.09	1	1	Multiple sclerosis
G40	13	0	89	177	279	0.05	1	0.67	Epilepsy and recurrent seizures
G45	4	1	29	151	185	0.03	0.8	0.84	Transient cerebral ischemic attacks and related syndromes
G47	1	0	3	47	51	0.02	1	0.94	Sleep disorders

G50	0	1	7	11	19	0.05	0	0.61	Disorders of trigeminal nerve
G61	1	0	5	9	15	0.07	1	0.64	Inflammatory polyneuropathy
G70	0	1	5	25	31	0.03	0	0.83	Myasthenia gravis and other myoneural disorders
G82	5	1	63	46	115	0.05	0.83	0.42	Paraplegia (paraparesis) and quadriplegia (quadriparesis)
G91	13	0	51	51	115	0.11	1	0.5	Hydrocephalus
G93	18	0	103	55	176	0.1	1	0.35	Other disorders of brain
G95	0	1	8	29	38	0.03	0	0.78	Other and unspecified diseases of spinal cord
I05	5	0	35	20	60	0.08	1	0.36	Rheumatic mitral valve diseases
I09	8	2	60	94	164	0.06	0.8	0.61	Other rheumatic heart diseases
I10	94	19	1348	3634	5095	0.02	0.83	0.73	Essential (primary) hypertension
I15	1	0	19	74	94	0.01	1	0.8	Secondary hypertension
I20	4	0	21	202	227	0.02	1	0.91	Angina pectoris
I21	5	0	69	53	127	0.04	1	0.43	STEMI & NSTEMI mocard infrc
I25	85	12	1007	2591	3695	0.03	0.88	0.72	Chronic ischemic heart disease
I26	1	0	6	29	36	0.03	1	0.83	Pulmonary embolism
I27	4	2	34	98	138	0.04	0.67	0.74	Other pulmonary heart diseases
I31	0	0	10	23	33	0		0.7	Other diseases of pericardium
I34	0	0	20	18	38	0		0.47	Nonrheumatic mitral valve disorders
I35	1	0	23	15	39	0.03	1	0.39	Nonrheumatic aortic valve disorders
I38	1	0	3	20	24	0.04	1	0.87	Endocarditis, valve unspecified
I46	3	0	13	6	22	0.14	1	0.32	Cardiac arrest
I47	1	0	4	76	81	0.01	1	0.95	Paroxysmal tachycardia
I48	7	1	50	114	172	0.05	0.88	0.7	Atrial fibrillation and flutter
I49	2	3	50	228	283	0.02	0.4	0.82	Other cardiac arrhythmias
I50	14	1	104	269	388	0.04	0.93	0.72	Heart failure
I51	4	0	68	71	143	0.03	1	0.51	Complications and ill-defined descriptions of heart disease

I60	13	0	75	100	188	0.07	1	0.57	Nontraumatic subarachnoid hemorrhage
I61	65	4	419	159	647	0.11	0.94	0.28	Nontraumatic intracerebral hemorrhage
I62	3	0	49	55	107	0.03	1	0.53	Other and unspecified nontraumatic intracranial hemorrhage
I63	69	6	755	1394	2224	0.03	0.92	0.65	Cerebral infarction
I65	1	1	14	46	62	0.03	0.5	0.77	Occls and stenosis of precerb art, not rslt in cereb infrc
I66	7	0	32	1	40	0.17	1	0.03	Occls and stenosis of cereb art, not rslt in cerebral infrc
I67	18	3	150	665	836	0.03	0.86	0.82	Other cerebrovascular diseases
I69	37	2	380	216	635	0.06	0.95	0.36	Sequelae of cerebrovascular disease
I70	1	0	35	121	157	0.01	1	0.78	Atherosclerosis
I71	3	0	26	29	58	0.05	1	0.53	Aortic aneurysm and dissection
I72	1	0	1	8	10	0.1	1	0.89	Other aneurysm
I74	1	0	2	0	3	0.33	1	0	Arterial embolism and thrombosis
I82	1	0	6	13	20	0.05	1	0.68	Other venous embolism and thrombosis
I85	1	0	9	0	10	0.1	1	0	Esophageal varices
I89	1	0	0	2	3	0.33	1	1	Oth noninfective disorders of lymphatic vessels and nodes
I97	1	0	0	0	1	1	1		Intraop and postproc comp and disorders of circ sys, NEC
J03	2	0	24	98	124	0.02	1	0.8	Acute tonsillitis
J06	2	2	65	370	439	0.01	0.5	0.85	Acute upper resp infections of multiple and unsp sites
J15	5	0	3	14	22	0.23	1	0.82	Bacterial pneumonia, not elsewhere classified
J18	100	15	406	1183	1704	0.07	0.87	0.74	Pneumonia, unspecified organism
J20	8	1	130	338	477	0.02	0.89	0.72	Acute bronchitis
J31	1	0	3	92	96	0.01	1	0.97	Chronic rhinitis, nasopharyngitis and

									pharyngitis
J33	1	0	3	39	43	0.02	1	0.93	Nasal polyp
J34	0	1	2	104	107	0.01	0	0.98	Other and unspecified disorders of nose and nasal sinuses
J38	1	0	5	62	68	0.01	1	0.93	Diseases of vocal cords and larynx, not elsewhere classified
J42	13	1	116	144	274	0.05	0.93	0.55	Unspecified chronic bronchitis
J43	8	1	89	49	147	0.06	0.89	0.36	Emphysema
J44	18	5	174	294	491	0.05	0.78	0.63	Other chronic obstructive pulmonary disease
J45	0	2	24	145	171	0.01	0	0.86	Asthma
J47	4	1	20	119	144	0.03	0.8	0.86	Bronchiectasis
J69	10	0	71	30	111	0.09	1	0.3	Pneumonitis due to solids and liquids
J70	1	0	5	0	6	0.17	1	0	Respiratory conditions due to other external agents
J80	1	0	12	1	14	0.07	1	0.08	Acute respiratory distress syndrome
J84	3	0	17	28	48	0.06	1	0.62	Other interstitial pulmonary diseases
J85	0	1	6	15	22	0.05	0	0.71	Abscess of lung and mediastinum
J86	2	0	5	9	16	0.13	1	0.64	Pyothorax
J93	4	0	32	27	63	0.06	1	0.46	Pneumothorax and air leak
J94	14	0	128	122	264	0.05	1	0.49	Other pleural conditions
J96	24	3	118	88	233	0.12	0.89	0.43	Respiratory failure, not elsewhere classified
J98	223	23	525	600	1371	0.18	0.91	0.53	Other respiratory disorders
K05	2	0	0	3	5	0.4	1	1	Gingivitis and periodontal diseases
K21	3	0	12	91	106	0.03	1	0.88	Gastro-esophageal reflux disease
K22	1	0	2	17	20	0.05	1	0.89	Other diseases of esophagus
K25	1	0	8	71	80	0.01	1	0.9	Gastric ulcer
K26	1	0	11	120	132	0.01	1	0.92	Duodenal ulcer
K29	3	1	43	549	596	0.01	0.75	0.93	Gastritis and duodenitis
K31	1	0	11	72	84	0.01	1	0.87	Other diseases of stomach and duodenum
K44	2	0	10	3	15	0.13	1	0.23	Diaphragmatic hernia

K52	1	0	2	39	42	0.02	1	0.95	Other and unsp noninfective gastroenteritis and colitis
K56	2	2	53	162	219	0.02	0.5	0.75	Paralytic ileus and intestinal obstruction without hernia
K60	1	0	1	112	114	0.01	1	0.99	Fissure and fistula of anal and rectal regions
K65	4	0	41	101	146	0.03	1	0.71	Peritonitis
K70	2	0	7	52	61	0.03	1	0.88	Alcoholic liver disease
K71	1	0	8	46	55	0.02	1	0.85	Toxic liver disease
K72	2	0	25	13	40	0.05	1	0.34	Hepatic failure, not elsewhere classified
K74	9	3	116	545	673	0.02	0.75	0.82	Fibrosis and cirrhosis of liver
K76	3	0	47	307	357	0.01	1	0.87	Other diseases of liver
K80	7	0	137	491	635	0.01	1	0.78	Cholelithiasis
K81	1	0	53	168	222	0	1	0.76	Cholecystitis
K82	0	0	37	114	151	0		0.75	Other diseases of gallbladder
K83	1	0	30	69	100	0.01	1	0.7	Other diseases of biliary tract
K85	7	0	72	102	181	0.04	1	0.59	Acute pancreatitis
K86	0	1	8	35	44	0.02	0	0.81	Other diseases of pancreas
K91	6	0	38	24	68	0.09	1	0.39	Intraop and postproc comp and disorders of dgstv sys, NEC
K92	11	1	101	207	320	0.04	0.92	0.67	Other diseases of digestive system
L08	1	0	10	26	37	0.03	1	0.72	Other local infections of skin and subcutaneous tissue
L40	1	0	4	123	128	0.01	1	0.97	Psoriasis
L80	1	0	0	17	18	0.06	1	1	Vitiligo
L89	3	0	26	26	55	0.05	1	0.5	Pressure ulcer
L97	1	0	3	1	5	0.2	1	0.25	Non-pressure chronic ulcer of lower limb, NEC
M06	4	1	63	106	174	0.03	0.8	0.63	Other rheumatoid arthritis
M13	1	0	27	153	181	0.01	1	0.85	Other arthritis
M19	1	0	0	2	3	0.33	1	1	Other and unspecified osteoarthritis
M32	1	1	65	41	108	0.02	0.5	0.39	Systemic lupus erythematosus (SLE)
M35	1	0	6	35	42	0.02	1	0.85	Other systemic involvement of

									connective tissue
M41	1	0	6	49	56	0.02	1	0.89	Scoliosis
M45	1	0	2	81	84	0.01	1	0.98	Ankylosing spondylitis
M47	1	1	38	360	400	0	0.5	0.9	Spondylosis
M50	1	0	45	368	414	0	1	0.89	Cervical disc disorders
M94	1	0	3	0	4	0.25	1	0	Other disorders of cartilage
N00	1	0	10	2	13	0.08	1	0.17	Acute nephritic syndrome
N04	3	1	11	229	244	0.02	0.75	0.95	Nephrotic syndrome
N13	0	0	48	249	297	0		0.84	Obstructive and reflux uropathy
N17	0	1	7	13	21	0.05	0	0.65	Acute kidney failure
N18	12	5	196	645	858	0.02	0.71	0.77	Chronic kidney disease (CKD)
N19	19	0	211	238	468	0.04	1	0.53	Unspecified kidney failure
N20	1	1	37	219	258	0.01	0.5	0.86	Calculus of kidney and ureter
N39	14	1	147	313	475	0.03	0.93	0.68	Other disorders of urinary system
N40	1	0	22	350	373	0	1	0.94	Enlarged prostate
N92	1	0	2	0	3	0.33	1	0	Excessive, frequent and irregular menstruation
P07	5	0	106	75	186	0.03	1	0.41	Disord of NB related to short gest and low birth weight, NEC
P15	1	0	5	0	6	0.17	1	0	Other birth injuries
P21	1	0	28	16	45	0.02	1	0.36	Birth asphyxia
P22	0	0	20	21	41	0		0.51	Respiratory distress of newborn
P23	10	0	273	4	287	0.03	1	0.01	Congenital pneumonia
P29	1	0	6	3	10	0.1	1	0.33	Cardiovascular disorders originating in the perinatal period
P59	1	0	44	90	135	0.01	1	0.67	Neonatal jaundice from other and unspecified causes
P96	1	0	2	0	3	0.33	1	0	Other conditions originating in the perinatal period
Q21	2	0	21	69	92	0.02	1	0.77	Congenital malformations of cardiac septa
Q24	1	1	44	102	148	0.01	0.5	0.7	Other congenital malformations of heart
Q28	1	0	5	2	8	0.13	1	0.29	Other congenital

									malformations of circulatory system
Q32	1	0	0	0	1	1	1		Congenital malformations of trachea and bronchus
R02	1	0	7	6	14	0.07	1	0.46	Gangrene, not elsewhere classified
R07	1	0	4	0	5	0.2	1	0	Pain in throat and chest
R09	1	0	1	6	8	0.13	1	0.86	Oth symptoms and signs involving the circ and resp sys
R18	2	0	15	48	65	0.03	1	0.76	Ascites
R27	2	0	6	0	8	0.25	1	0	Other lack of coordination
R31	1	0	2	31	34	0.03	1	0.94	Hematuria
R33	3	0	29	7	39	0.08	1	0.19	Retention of urine
R40	1	0	0	1	2	0.5	1	1	Somnolence, stupor and coma
R57	9	1	38	40	88	0.11	0.9	0.51	Shock, not elsewhere classified
R68	4	1	11	4	20	0.25	0.8	0.27	Other general symptoms and signs
R90	2	0	11	38	51	0.04	1	0.78	Abnormal findings on diagnostic imaging of cnsl
R91	1	0	20	104	125	0.01	1	0.84	Abnormal findings on diagnostic imaging of lung
S01	2	0	9	7	18	0.11	1	0.44	Open wound of head
S02	6	1	48	156	211	0.03	0.86	0.76	Fracture of skull and facial bones
S06	45	4	380	332	761	0.06	0.92	0.47	Intracranial injury
S09	1	0	23	89	113	0.01	1	0.79	Other and unspecified injuries of head
S12	2	1	23	45	71	0.04	0.67	0.66	Fracture of cervical vertebra and other parts of neck
S14	1	0	19	28	48	0.02	1	0.6	Injury of nerves and spinal cord at neck level
S22	1	0	78	182	261	0	1	0.7	Fracture of rib(s), sternum and thoracic spine
S27	4	1	35	48	88	0.06	0.8	0.58	Injury of other and unspecified intrathoracic organs
S29	1	0	11	32	44	0.02	1	0.74	Other and unspecified injuries of thorax
S32	4	1	118	313	436	0.01	0.8	0.73	Fracture of lumbar spine and pelvis

S39	1	0	19	15	35	0.03	1	0.44	Oth & unsp injuries of abd, low back, pelv & extrn genitals
S42	0	0	45	265	310	0		0.85	Fracture of shoulder and upper arm
S72	7	1	200	454	662	0.01	0.88	0.69	Fracture of femur
S82	3	0	138	538	679	0	1	0.8	Fracture of lower leg, including ankle
T02	3	2	24	38	67	0.07	0.6	0.61	Fractures involving multiple body regions
T07	3	0	46	102	151	0.02	1	0.69	Unspecified multiple injuries
T09	1	0	17	16	34	0.03	1	0.48	Other injuries of spine and trunk, level unspecified
T29	2	1	54	148	205	0.01	0.67	0.73	Burns and corrosions of multiple body regions
T60	1	0	2	1	4	0.25	1	0.33	Toxic effect of pesticides
T79	1	0	4	3	8	0.13	1	0.43	Certain early complications of trauma, NEC
T79	1	0	4	3	8	0.13	1	0.43	Certain early complications of trauma, NEC
T79	1	0	4	3	8	0.13	1	0.43	Certain early complications of trauma, NEC
T79	1	0	4	3	8	0.13	1	0.43	Certain early complications of trauma, NEC
T86	3	0	25	36	64	0.05	1	0.59	Complications of transplanted organs and tissue
T90	2	0	30	33	65	0.03	1	0.52	Sequelae of injuries of head
X59	0	0	11	33	44	0		0.75	Exposure to specified factors
Z08	6	1	66	132	205	0.03	0.86	0.67	Encounter for follow-up examination after completed treatment for malignant neoplasm
Z93	2	0	12	8	22	0.09	1	0.4	Artificial opening status
Z94	16	0	151	100	267	0.06	1	0.4	Transplanted organ and tissue status
Z95	1	0	5	39	45	0.02	1	0.89	Presence of cardiac and vascular implants and grafts
Z98	1	1	7	41	50	0.04	0.5	0.85	Other postprocedural

states
